# Biosecurity Threat Posed by Botulinum Toxin

**DOI:** 10.3390/toxins11120681

**Published:** 2019-11-20

**Authors:** Orlando Cenciarelli, Paul William Riley, Agoritsa Baka

**Affiliations:** European Centre for Disease Prevention and Control (ECDC), Gustav III:s Boulevard 40, 169 73 Solna, Sweden

**Keywords:** botulinum, botulism, biosecurity, bioterrorism, biocrimes, poisoning.

## Abstract

The deliberate release of biological agents with terrorist or criminal intent continues to pose concerns in the current geopolitical situation. Therefore, attention is still needed to ensure preparedness against the potential use of pathogens as unconventional weapons. Botulinum neurotoxin (BoNT) is one such biological threat, characterized by an extremely low lethal dose, high morbidity and mortality when appropriately disseminated, and the capacity to cause panic and social disruption. This paper addresses the risks of a potential release of the botulinum neurotoxin and summarizes the relevant aspects of the threat.

## 1. Introduction

Botulinum neurotoxin (BoNT), in its purified form, is the most toxic substance known. A double-chain protein with a molecular weight of 150 kDa, it exists in seven different serotypes (A to G) and more than 40 subtypes. In the past few years, using bioinformatics, data mining and high-throughput sequencing techniques, other subtypes of BoNT have been discovered, as well as new BoNT-like toxins produced by members of non-clostridial bacterial species (e.g., *Enterococcus faecium* and *Chryseobacterium piperi*) [1,2].

BoNTs are zinc proteases produced by several members of the genus *Clostridium*, including *C. argentinense*, *C. baratii*, *C. butyricum* and *C. sporogenes,* but mostly by *C. botulinum*–an anaerobic, Gram-positive, spore-forming bacillus widely found in soils [3,4]. Botulism presents in six recognised clinical forms: foodborne, infant, intestinal, wound, iatrogenic, and inhalational, each characterised by different exposure routes and incubation periods between contamination and the onset of symptoms [4] (Table 1). Botulism in humans is usually associated with serotypes A, B, E and F [5].

The active form of BoNT is composed of a heavy chain linked to a light chain via a disulfide bond. The light chain is the active part of the toxin and has proteolytic activity. BoNT binds selectively and irreversibly to nerve terminals and prevents neurotransmission by inhibiting the release of acetylcholine, resulting in flaccid paralysis.

The molecular mechanism of action of BoNT involves blocking the release of the neurotransmitter acetylcholine to the nerve terminals, through the cleaving of one of the SNARE (“Soluble NSF Attachment Protein Receptor”) proteins (i.e., VAMP/Synaptobrevin, Syntaxin and SNAP-25). SNARE proteins are responsible for the fusion of the synaptic vesicle (containing the neurotransmitter) with the presynaptic plasma membrane [16,17].

The clinical symptoms of botulism are independent of the route of contamination. The hallmark clinical syndrome is described as afebrile descending symmetric paralysis, starting from the cranial nerves (typical symptoms: diplopia, dysphagia, dysarthria, dry mouth), followed by weakness and dyspnoea due to the paralysis of intercostal respiratory muscles and the diaphragm. Patients may exhibit nausea and vomiting before paralysis. Throughout the symptom evolution, patients remain oriented, as BoNT cannot cross the blood–brain barrier [5].

When subjected to stress (e.g., starvation, low salt and sugar concentrations), and under specific environmental conditions (e.g., anaerobic environment and basic pH), *C. botulinum* is able to form spores which are extremely resistant to environmental factors (e.g., temperature, chemicals) and can remain viable for many years [18]. In contrast to the spores, BoNT is sensitive to high temperatures and to common disinfectants; both heating to 85 °C for 5 min [15,19] or using a 0.1% hypochlorite solution [20] are enough to degrade the toxin. The LD_50_ for BoNT is extremely low in humans, as has been inferred from primate studies (Table 2).

**Figure 1:**
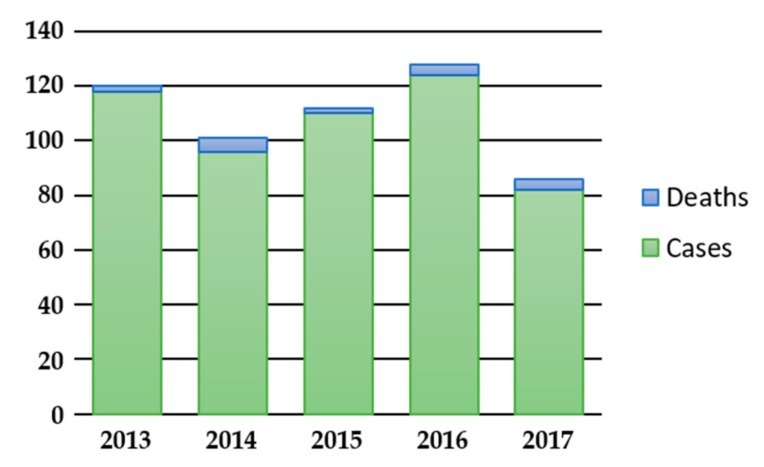
Botulism cases and deaths reported in the EU/EEA in 2013–2017 [22].

As a naturally occurring disease, botulism is rather rare. In Europe, in the five-year period from 2013 to 2017, 547 confirmed cases and 17 deaths were reported by 22 EU/EEA Member States, with an average number of 109 cases per year (min. 86 cases in 2017, max. 128 cases in 2016) (Figure 1) [22]. The largest number of cases during this period were reported by Italy (130), Romania (84), Poland (74) and France (58), with clusters associated with the consumption of specific food vehicles [23]. During the same five-year period, 900 confirmed cases and 15 deaths were reported in the USA, with an average number of 180 cases per year (min. 153 cases in 2013, max. 205 cases in 2016) [24].

The diagnosis of botulism involves the clinical presentation and laboratory identification of BoNTs in serum and other clinical specimens (vomitus, gastric aspirate, nasal swabs or stool) [11,25,26,27], or in suspected food [28]. The mouse bioassay (MBA) is still considered to be the gold standard for laboratory testing due to its high sensitivity [27]. However, this method is costly, labor intensive and time-consuming, requires trained personnel and has ethical implications due to the use of live animals [29]. In cases of inhalational botulism, BoNT would not be detectable in serum or stool samples and the best approach then would be an ELISA test on nasal or broncho-alveolar lavage within 24 h of inhalation [28]. The differential diagnosis of botulism includes a number of neuromuscular diseases or central nervous system disorders, including cerebrovascular accident (CVA), myasthenia gravis, Lambert–Eaton syndrome, tick paralysis, Guillain–Barré syndrome, poliomyelitis, brainstem stroke, and heavy metal poisoning [26,27,30].

The treatment of patients with suspected botulism involves supportive care (e.g., mechanical ventilation, hydration, prevention of secondary infections, etc.) and the use of botulism antitoxin (BAT) I.V., ideally within 72 h of the onset of symptoms. In the EU/EEA the trivalent (A, B, E; equine) [28,31] antitoxin and supportive treatments are used. In 2013, a human-derived BoNT antitoxin (BIG-IV) for the treatment of infant botulism was licensed in the U.S. by the FDA (BabyBIG™). This formulation has been made available for the treatment of patients under one year of age hospitalized outside the U.S since 2005 [32]. The prompt administration of the antitoxin is important, as only circulating BoNT can be neutralised. The antitoxin becomes ineffective when the BoNT enters the nerve terminals. The combination of supportive therapy and the administration of the antitoxin can stop the progression of clinical symptoms [25]; however, supportive care—especially mechanical ventilation—is required until new synapses grow, i.e., for 2–4 months.

In the last few years, small molecules have been found which are able to inhibit the effects of BoNT and have been proven to be non-toxic in MBA. Arzania Tehran et al., in 2015 [33], have shown the effectiveness of EGA (2-(4-bromobenzylidene)-N-(2,6-dimethylphenyl) hydrazinecarboxamide) in vitro, in preventing the toxicity of BoNTs by interfering with the toxin trafficking mechanism. The study demonstrated that in vivo, EGA is able to reduce the symptoms after intoxication with BoNT/A, and reduce mortality after intoxication with BoNT/B and BoNT/D [33]. Pirazzini and Rossetto [34] describe the possible molecular targets for the development of putative pan-inhibitor drugs targeting a step of BoNT intoxication common to all the serotypes and subtypes. The authors identified the following stages as possible targets for a non-serotype and non-subtype specific toxin inhibitor: (i) neutralization of extracellular toxin; (ii) inhibition of toxin binding; (iii) inhibition of toxin internalization and trafficking; (iv) inhibition of toxin translocation by (a) blocking of endocytic vesicle acidification, (b) blocking of HC channel formation and (c) blocking of light chain refolding; (v) inhibition of the toxin disulfide bond reduction; (vi) inhibition of SNARE cleavage by the light chain through non-peptidic or peptidic light chain inhibitors; (vii) reversion of BoNT paralysis through intracellular toxin stability or neuromuscular junction recovery [34].

There is currently no licensed vaccine to protect against botulism. In the past, an inactivated pentavalent botulinum toxoid (PBT) vaccine (A-E) [35] was used in the United States for the vaccination of persons at higher risk of contracting the disease, such as personnel of laboratories, research and manufacturing facilities. However, its use was discontinued in 2011, after a review of available data indicated a decline in the immunogenicity of some of the toxin serotypes and the occurrence of moderate local reactions related to annual boosters [36,37]. There are several candidate vaccines to replace the PBT vaccine, including recombinant protein-based, DNA-based and viral vector-based vaccines [37].

## 2. Biowarfare, Bioterrorism and Biocrimes

Biological agents (BAs) have been used for biowarfare (BW) and bioterrorism (BT)/biocriminal (BC) purposes since ancient times. In these contexts, there is a certain attractiveness of using BAs over conventional (e.g., explosives) or other unconventional (i.e., chemicals or radiological/nuclear) agents. It lies in the relatively lower cost of production in comparison to conventional agents [38], the very low LD_50_–especially for toxins–compared with chemical agents [39], and, consequently, the very small quantities required to affect large numbers of people. Moreover, some of the BAs of concern are also transmissible person-to-person, or are difficult to diagnose (due to symptoms that can mimic the early phase of common, naturally occurring diseases, e.g., influenza-like illnesses or gastroenteritis). In the case of botulism, there is no person-to-person transmission; however, treatment of more than a limited number of patients would put a significant strain on the health system due to the high demand of critical care and low availability of antitoxin. Factors that inhibit the use of BAs include the fact that the acquisition or production of a sufficient quantity in purified form is difficult, and that effective dissemination methods are limited and unpredictable [38].

Various lists of pathogens of concern have been published by various groups, including the European Commission (EC) [40] and the US Centers for Diseases Control and Prevention (CDC) [41]. Generally, the same groups of pathogens are featured in all of them with some variations. BoNT is included in them all, as it displays a lot of the suggested characteristics of a high-threat agent.

## 3. BoNT as a Weapon

The literature reports a number of attempts to produce and use BoNT as a bioweapon in the past decades. Studies on the potential use of the BoNT as a BA for biowarfare purposes date back to the 1930s at Unit 731—a biowarfare research unit of the Japanese Army in Manchuria [42]. The lead scientist of the program admitted to having fed prisoners with cultures of *C. botulinum*, with lethal effects.

Cases of use, possession or interest in the acquisition of BoNT have been reported over the last decades for both criminal and terrorist purposes [43]. The best-known example was perpetrated by the Japanese cult Aum Shinrikyō, which attempted to use BoNT on three different occasions during the period April 1990–March 1995. The cult obtained *C. botulinum* from environmental soil samples and used BoNT at least three times, with the first instance taking place in April 1990, when three vehicles disseminated BoNT close to strategic targets in the Tokyo area (the Japanese parliament, the town of Yokohama, the Yokosuka U.S. Navy base, and the Narita International Airport); the second, in June 1993, on the day of Prince Naruhito of Japan’s wedding, using a specially equipped vehicle in downtown Tokyo; thirdly, in March 1995, when three briefcases designed to release BoNT were left in the Tokyo subway [43]. According to Carus (2001) [43], all the attacks failed due to errors made in the production of the toxin (i.e., poor laboratory techniques) or in dissemination (i.e., incorrect dispersion methods). Other reasons for the failure of the attacks were reported to be the chosen strain of *C. botulinum*, and/or the small amounts of BoNT disseminated.

BoNT meets several of the criteria listed in the previous section to qualify as a BA with the potential to be used for offensive purposes: the LD_50_ of BoNT is lower than that of any other substance known; the toxin can be disseminated via different routes (inhalational or oral, but also injection), and the symptoms can be misinterpreted in the initial stages. Moreover, *C. botulinum* is widespread present in soil, making its acquisition from natural sources possible, and the vegetative form, the spores or the toxin can be easily transported. Finally, BoNT in solution is colourless, odourless and tasteless, making the BA ideal for a silent attack. The intentional release of BoNT would have a major impact on public health, due to the rather short incubation period ranging from hours to few days, depending on the route and the amount of toxin adsorbed (this can slow recognition of an intentional release), the potency and mortality of the untreated disease, and the need for intensive and long-term healthcare support for the victims (e.g., ventilation) [21]. The disadvantages of using BoNT as a BA include the complexity of the toxin purification process for a large-scale attack, both in terms of the technology required and the potential risk of exposure to the preparer, and the high decay rate of the toxin in the environment. Below, we consider the main potential dissemination routes for BoNT, and the feasibility of carrying out an attack using each specific route.

### 3.1. BoNT as an Injectable Preparation

The use of an injectable BoNT is not a likely choice for a large-scale attack, unless the BoNT is used to contaminate injectable substances (e.g., vaccines, medications, etc.) [44]. However, this approach is unlikely, due to the need to produce and purify a significant amount of toxin, and the need to overcome the safety and security procedures during industrial production, packaging and distribution of injectable substances. However, the use of BoNT in small-scale attacks or targeted assassinations (i.e., for BC purposes) cannot be excluded.

BoNT has a number of applications in therapeutics for a wide range of clinical and aesthetic conditions, from blepharospasm or torticollis, to muscle spasticity or migraine [45]. An experiment carried out on non-human primates in 1988 [46], found that the intramuscular LD_50_ for one commercial formulation of BoNT was approximately 40 Units/kg. Extrapolated to humans, the LD_50_ for an individual of 70 kg is approximately 2800 Units. Commercially available BoNT (e.g., Botox^®^, Xeomin ^®^ or Dysport^®^) contains between 100 and 500 Units/vial [47], therefore the use of commercial forms of BoNT for BC, even if limited due to accessibility (i.e., the need for a medical prescription), cannot be excluded. Access to pharmaceutical products using the internet increases the risk of medical–cosmetic BoNT being used for offensive purposes. The commercialisation of counterfeit products from internet sources is of particular concern, due to the fact that the amount of BoNT contained in these preparations can bear no relation to the amount declared on the label [48]. Finally, the production of BoNT for counterfeit products in unauthorised facilities poses serious security risks, due to the increased accessibility for terrorists to large amounts of the toxin that could be used for other routes of contamination.

### 3.2. Food and Beverage Contamination with BoNT

The contamination of food and beverages with BoNT represents one potential route for an intentional attack; however, as with the contamination of medical and cosmetic preparations, the feasibility of using this route is significantly limited. The large-scale contamination of commercial food or beverages requires access to production, packaging or distribution facilities. More importantly, BoNT is subject to degradation in food due to its chemical characteristics (e.g., the pH, the sugar content, etc.), and the physical treatments food is subjected to during preparation (i.e., temperature changes). High temperatures, low pH and high sugar content all contribute to a rapid degradation of the BoNT [49]. The contamination of small amounts of food sold for immediate consumption without further cooking can be a potentially more effective approach, especially considering the naturally occurring, small clusters of botulism cases from home-prepared canned food or dried fish [23].

BoNT has been reported to be quite stable in some fluids. In 1995, Kazdobina reported BoNT retains 50% of its initial toxicity for up to 70 days in beer, mineral and fruit waters, dessert wine and 40° alcoholic beverages [50]. According to these findings, the intentional contamination of beverages appears to be more feasible. The intentional contamination of water supply networks is another scenario which is considered unlikely, due to the inactivation of BoNT caused by chlorine used for water treatment, but also its degradation from hydrolysis by exposure to air and ultraviolet radiation from sunlight. The contamination of a water reservoir would require a relatively large amount of BoNT which, in addition, would need to be timed to coincide with human consumption.

Wein and Liu, in 2005 [51], analysed the intentional contamination of a milk supply chain with BoNT using a model, taking into consideration the effects of milk processing. Their model suggests that the contamination of a milk supply chain could lead to the poisoning of more than 500,000 people, if 10 g of BoNT was used to contaminate the milk at one of the following stages of the milk supply chain: in a holding tank at a dairy farm; in a tanker truck transporting milk from a farm to the processing plant; or in a raw milk silo at the processing facility.

### 3.3. Airborne Release of BoNT

The airborne release of BoNT is one more likely scenario for an attack. A point-source aerosol release of the purified toxin has been estimated to be able to kill 10% of exposed individuals within a distance of 500 m downwind [21]. The airborne release scenario also has several limitations, mostly due to the stability of BoNT in the environment and the persistence of the toxin at the site of release. These factors strictly depend on the purity of the prepared BoNT and the size of the aerosol particles, as well as on environmental and weather conditions. Villar and colleagues [49] reported that BoNT degraded under sunlight within 1 to 3 h, with an estimated decay rate of between 1% and 4% per minute, dependent on weather conditions (e.g., temperature, humidity) and the dispersal pattern.

## 4. Indicators of an Intentional Release of BoNT

Due to botulism occurring naturally worldwide, covert attacks using BoNT cannot be immediately suspected [52]. However, botulism is rather rare—any outbreak involving more than one case with clinical symptoms of acute flaccid paralysis should be carefully investigated, considering also the hypothesis of an intentional release.

Surveillance, environmental and epidemiologic investigations are of considerable importance in the identification of potential intentional releases of BAs, including BoNT. Specific indicators for an intentional release of BoNT include the following:

(1) An unusual distribution or clustering of the cases (e.g., several patients who attended the same event or that visited the same place (e.g., an airport or a subway station) at the same time, but who did not eat the same food).
(2) A large number of cases associated with the consumption of industrially prepared food. As naturally occurring botulism is mostly associated with home-made food, an outbreak linked to commercial food could point to the intentional release of BoNT.
(3) An outbreak associated with an uncommon BoNT type.
(4) Several outbreaks that do not have immediately identifiable common food or geographical point sources.

## 5. Discussion and Conclusions

Every case of botulism requires careful epidemiological investigation, due to the need to identify a potential common food-related source as soon as possible and eliminate the risk of further exposure for more persons.

The BoNT has several characteristics that make it a good candidate agent for an intentional release: it is odourless, colourless, tasteless, and can be spread through different routes. On the other hand, its production, purification, storage, transportation, and dispersion can prove difficult, due to the intrinsic characteristics of the agent, especially if the process is performed by poorly trained scientists or in sub-standard laboratory settings.

An intentional release of BoNT (either through foodstuff contamination or airborne release) would have a significant impact on public health, resulting in significant morbidity and mortality of the exposed persons, causing widespread panic and the disruption of the health system. As is the case with all BAs of very high threat, this highlights the continued need to be vigilant and prepared. Furthermore, the recent findings on new types and subtypes of BoNT, as well as new evidence of BoNTs and BoNT-related genes in other bacterial genera besides the *Clostridia*, underline the importance of continuing to deepen research to understand the physiology and evolution of BoNT and BoNT-producing bacteria.

## Figures and Tables

**Table 1:** Main characteristics of the different clinical forms of botulism.

Clinical Form	Source of Contamination	Incubation Period from Contamination to onset of Symptoms
**Foodborne botulism**	Mainly caused by the ingestion of home-preserved food containing BoNT, more rarely by the ingestion of a mixture of BoNT, vegetative cells and spores	4 h to 10 days (typically 8–36 h) [6]
**Infant botulism**	Caused by the ingestion of spores in infants of 1 week to 12 months of age (typically 1 to 6 months of age)	3 to 30 days [7]
**Intestinal botulism**	Caused by the ingestion of spores in children older than 12 months of age and in adults [8,9]	Unknown
**Wound botulism**	Caused by spores that germinate in a wound; quite often associated with drugs injections which cause skin disruption and provide an environment for the production of BoNT [10]	7 to 14 days [11,12]
**Iatrogenic botulism**	Caused by the injection of commercial or non-approved BoNT preparations [13]	Unknown
**Inhalational botulism**	Caused by the inhalation of BoNT; the toxin enters the circulatory system through mucosal membranes. This is not a natural route of exposure, and it has been described only as an accidental laboratory exposure [4]	24–36 h to several days [14,15]

**Table 2:** Estimated LD_50_ for BoNT/A for a human weighing 70 kg [21].

Route	LD_50_
Intravenous/intramuscular	0.09–0.15 µg
Inhalation	0.70–0.90 µg
Ingestion	70 µg
